# Artificial Intelligence in Global Epidemics, Part 2: Editorial

**DOI:** 10.1007/s00354-022-00196-w

**Published:** 2022-11-23

**Authors:** Gurdeep Singh Hura, Sven Groppe, Sarika Jain, Le Gruenwald

**Affiliations:** 1 University of Maryland Eastern Shore, Princess Anne, MD USA; 2 University of Lübeck, Lübeck, Germany; 3 National Institute of Technology, Kurukshetra, India; 4 University of Oklahoma, Norman, OK USA

## Introduction

**Fig. 1:**
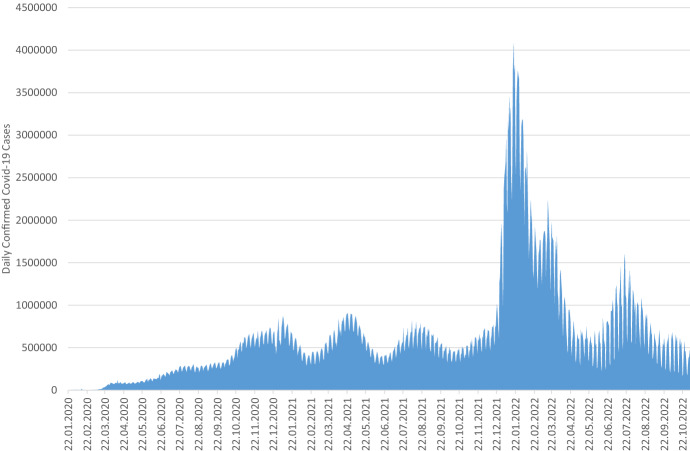
Daily COVID-19-confirmed cases worldwide (Data source: Johns Hopkins University CSSE COVID-19 Data)

**Fig. 2:**
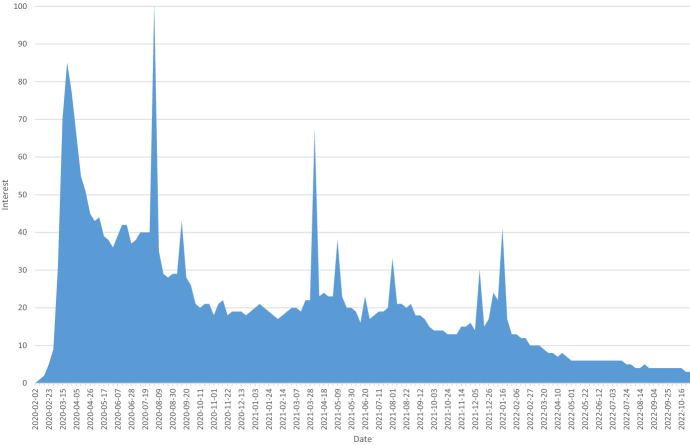
Interest in the search term “covid-19” in Google trends using https://trends.google.de/trends/explore?date=2019-05-10%202022-11-05 &q=covid-19 (visited on 5.11.2022)

**Fig. 3:**
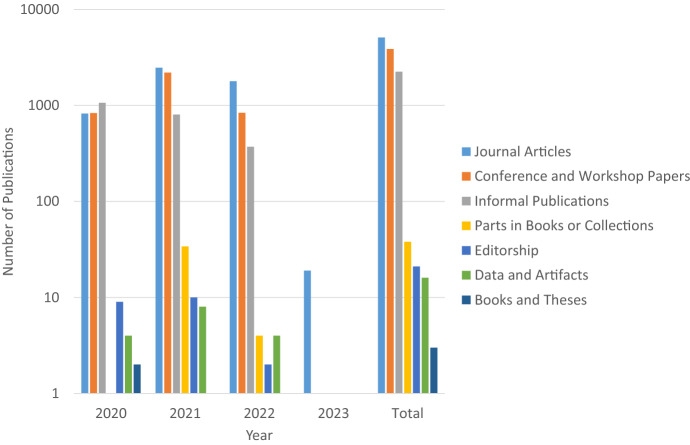
Number of Publications versus year in DBLP searching for “covid-19” using https://dblp.org/search/publ?q=covid-19 (visited on 5.11.2022)

Although the number of confirmed COVID-19 cases is still relatively high (see Figure 1), the interest of the general public in the COVID-19 pandemic is almost coming to an end (see Figure 2). One might expect an analogous trend for the interest of researchers in the COVID-19 pandemic. However, the availability of preliminary data and previous research results as the basis of current and future research seems to encourage researchers to provide more useful data at global level. It looks quite obvious that the results of the current research in the area of COVID-19 are published in numerous scientific contributions in recent times (see Figure 3). We expect this number will continue to rise, because the year 2022 is not over yet and it takes some time until bibliographic databases add the metadata of recent publications. Furthermore, in 2020, informal publications were the dominant form of publication promising a fast publication process; this has supported timely discussions among researchers about the new COVID-19 illness for fast medical solutions. Since 2021, peer-reviewed publications like journal articles, which are known for more mature and productive research results, have dominated.

Because of this large number of continuous research results, our special issue also received a high number of submissions, such that the included articles have been too many for only one issue. Hence, this special issue has been divided into two parts, where Part 1 was already published in 2021 [7]. Part 2 with 13 articles [1–6, 8–14] is introduced in this editorial. Later publications about COVID-19 will be included in the regular issues of the New Generation Computing journal.

This special issue solicits submissions focusing on the perspectives and surveys of existing scenarios and consisting of research results in the following, but not limited to, topics:

- Effects of COVID-19 Confinements
- Assessing Countries’ readiness for coping with epidemics
- Studies related to animal originated diseases
- Challenges in battling with epidemics
- AI for forecasting
- AI and detection
- Generating recommendations
- AI in genome sequencing
- AI-assisted testing
- Role of AI in contact tracing
- Situation awareness
- Computational drug repurposing

## Selected Papers

The topics of our selected papers are many fold. While many contributions focus on image processing and detection of COVID-19 [3, 8, 11, 14], there is also a shift to multi-disease prediction [10] and predicting post-COVID-19 complications [5], which have not been addressed in research in the early days of COVID-19.

Sentiment analysis [1, 13] continues to be a topic of interest as it was in the times of the COVID-19 pandemic. Not only the data from social media [13] is used, but also the data from other data sources like hospital reviews [1], which is a sign of the availability of more and more different types of data sources and hence better possibilities for research.

Ideas in the context of COVID-19 are nowadays widespread targeting mobility aspects [2, 9], evaluation of information relevance [12], introducing advanced intelligent cyber-physical healthcare framework [6] and forecasting COVID-19 reliability of the countries [4].

We hope you enjoy reading the papers and help you to understand the current status of COVID-19-related research. We also hope these papers will provide you with some directions for research in this area. We believe that COVID-19 will remain a major focus for further research and experimentation in the times to come.
